# Balancing Speed and Cost: Economic Insights from Rapid Diagnostic Testing in Bloodstream Infections

**DOI:** 10.3390/antibiotics15030320

**Published:** 2026-03-20

**Authors:** Gergana Lengerova, Ralitsa Raycheva, Michael M. Petrov, Todor Kantardjiev

**Affiliations:** 1 Department of Medical Microbiology and Immunology “Prof. Dr. Elissay Yanev”, Medical University Plovdiv, 15A V. Aprilov Blvd., 4002 Plovdiv, Bulgaria; michael.petrov@mu-plovdiv.bg (M.M.P.);; 2 Microbiology Laboratory, St. George University Hospital, 15A V. Aprilov Blvd., 4002 Plovdiv, Bulgaria; 3 Research Institute at Medical University Plovdiv, 15A V. Aprilov Blvd., 4002 Plovdiv, Bulgaria; 4 Department of Social Medicine and Public Health, Medical University Plovdiv, 15A V. Aprilov Blvd., 4002 Plovdiv, Bulgaria; r.raycheva@mu-plovdiv.bg

**Keywords:** bloodstream infection, rapid diagnostic testing, MALDI-TOF MS, multiplex PCR, fluorescence in situ hybridization, antimicrobial stewardship, cost-effectiveness, hospital costs

## Abstract

**Background:** Rapid diagnostic tests (RDTs) for bloodstream infections (BSIs) reduce time to pathogen identification, yet evidence on their real-world economic and clinical value remains inconsistent. This study aimed to compare clinical outcomes, antibiotic utilization, and hospital costs associated with different rapid microbiological identification methods versus standard culture. **Methods:** A retrospective observational study was conducted in a tertiary university hospital including 115 hospitalized patients with suspected or confirmed BSIs. Multiplex PCR (mPCR), fluorescence in situ hybridization (FISH), and MALDI-TOF MS were compared with conventional culture. Outcomes included mortality, length of stay, antibiotic-days, and direct and indirect hospital costs. Nonparametric and exploratory adjusted analyses were performed. **Results:** No significant differences were observed across diagnostic groups for age, sex, mortality, or length of stay. Patients tested with mPCR showed higher empirical and total antibiotic-days and increased antibiotic-related costs (*p* < 0.05). Median direct and indirect hospital costs were numerically lower with FISH and mPCR but did not reach statistical significance. Adjusted analyses confirmed that diagnostic modality was not independently associated with mortality or costs. **Conclusions:** Rapid diagnostics accelerate identification but demonstrate heterogeneous downstream clinical and economic effects. Their value appears to depend more on local implementation and antimicrobial stewardship integration than on diagnostic speed alone.

## 1. Introduction

Bloodstream infections (BSIs) are a major source of morbidity, mortality, and healthcare expenditure in hospitalized patients worldwide. Timely and accurate identification of causative pathogens is critical for optimizing antimicrobial therapy and improving patient outcomes, as delays in appropriate treatment are associated with increased mortality and prolonged hospital stays [1,2]. Conventional blood culture remains the gold standard for BSI diagnosis but is limited by lengthy turnaround times and susceptibility to false negatives, particularly in patients receiving prior antimicrobial therapy or with polymicrobial infections [1,3].

Recent advances in rapid microbiological identification methods—including nucleic acid amplification tests, matrix-assisted laser desorption ionization–time-of-flight mass spectrometry (MALDI-TOF MS), and multiplex PCR panels—have enabled pathogen detection and resistance marker identification within hours, substantially reducing time to targeted therapy [4,5,6,7,8]. The American Society for Microbiology, in its evidence-based laboratory medicine guidelines, strongly recommends the use of rapid diagnostic tests combined with active communication to decrease time to targeted therapy and hospital length of stay in patients with BSIs [4]. However, randomized trials and meta-analyses indicate that while rapid diagnostics consistently accelerate optimal antimicrobial initiation, their impact on mortality, length of stay, and overall healthcare costs is variable and may depend on integration with antimicrobial stewardship programs [9,10,11,12,13].

Economic evaluations suggest that certain rapid diagnostic strategies, particularly when paired with stewardship interventions, can be cost-effective and may yield substantial savings by averting deaths and reducing resource utilization [9,12,13]. Nevertheless, large multicenter trials have reported modest differences in cost and survival, highlighting the need for context-specific analyses and consideration of real-world implementation factors [10,11]. This study aims to systematically compare the cost and clinical outcomes of rapid microbiological identification methods versus standard culture in hospitalized patients with BSIs, addressing critical gaps in the evidence base and informing best practices for diagnostic stewardship and resource allocation.

## 2. Results

### 2.1. Patient Demographics and Clinical Characteristics

A total of 115 patients were included in the study (69.6% male), with a median age of 52 years (33, 62). No statistically significant differences were observed between the two diagnostic groups regarding age (U = 1288, *p* = 0.298) or sex (z = 1.1, *p* = 0.267) (Table 1).

### 2.2. Comparative Analysis of Clinical and Economic Outcomes Between Rapid Diagnostic Tests and Standard Culture

A subgroup comparative analysis was performed between rapid diagnostic tests and the standard culture-based microbiological method. The results are summarized in Table 1. No statistically significant differences were observed by sex (χ^2^ = 3.839, df = 3; *p* = 0.279) or age (H = 7.042; *p* = 0.071). The median number of hospital days was similar across the four study groups (H = 7.028; *p* = 0.071) (Table 1).

A significant difference was found in the median duration (antibiotic-days) of empirical antibiotic therapy (H = 13.54; *p* = 0.004), with this value being statistically significantly higher in the mPCR group compared to each of the other groups—FISH (*p* = 0.001), MALDI-TOF (*p* = 0.002), and standard culture (*p* = 0.021)—but not for targeted therapy (H = 3.98; *p* = 0.263). As expected, a difference was also demonstrated in the total duration (antibiotic-days) of antibiotic therapy (H = 10.90; *p* = 0.012). Pairwise comparisons showed that the median total antibiotic-days in patients from the mPCR group were significantly higher compared to those diagnosed by FISH (*p* = 0.005) and standard culture (*p* = 0.003) (Table 1).

These results were reproduced with respect to both the costs of empirical antibiotic therapy (H = 7.95; *p* = 0.047) and the total antibiotic therapy costs (H = 10.34; *p* = 0.016). In pairwise comparisons, the median costs for (1) empirical antibiotic therapy and (2) total antibiotic therapy were significantly higher in patients diagnosed using mPCR compared with the following: FISH—(1) *p* = 0.008, (2) *p* = 0.011; MALDI-TOF—(1) *p* = 0.010; and standard culture—(1) *p* = 0.007, (2) *p* = 0.018 (Table 1).

### 2.3. Additional Subgroup Analyses: Antibiotic Utilization, Mortality, and Cost Patterns

To explore the obtained results in greater depth, several additional subgroup analyses were conducted. First, the ratio of antibiotic-days to hospital days was examined for each study group. No statistically significant differences were observed among the groups (H = 4.45; *p* = 0.217). Nevertheless, the median ratio was highest in the mPCR group (1.80), indicating that the number of antibiotic-days in this group was almost twice the number of hospital days. This finding helps explain the statistically significant differences in antibiotic-days and related costs described above. For comparison, the ratio values in the other groups were as follows: FISH (1.6), MALDI-TOF (1.4), and standard culture (1.2) (Table 1).

No statistically significant association was found between the type of diagnostic test and mortality (χ^2^ = 1.42, df = 3; *p* = 0.701). None of the rapid diagnostic tests resulted in a significantly reduced risk of death: mPCR: OR = 0.87, 95% CI 0.32–2.35 (z = 0.27, *p* = 0.786); FISH: OR = 1.44, 95% CI 0.51–4.06 (z = 0.69, *p* = 0.49); MALDI-TOF: OR = 0.75, 95% CI 0.27–2.06 (z = 0.56, *p* = 0.573). The relatively high mortality rates observed across groups reflect the critically ill ICU population included in the study, characterized by severe or very severe clinical conditions and suspected or confirmed bloodstream infections. Additionally, the distribution of hospital days was analyzed according to patient condition severity, with subsequent therapeutic action taken following the diagnostic result, outcome, and diagnostic method (four groups). The distribution of patients by condition severity and diagnostic group was as follows: (1) mPCR: severe 33.33%, very severe 66.77%; (2) FISH: severe 40.00%, very severe 60.00%; (3) MALDI-TOF: severe 24.00%, very severe 76.00%; (4) standard culture: severe 39.50%, very severe 60.50%. The lowest median number of hospital days was observed among patients diagnosed by FISH (20 days) and mPCR (23.5 days), followed by MALDI-TOF (28 days) and standard culture (34 days) (H = 3.34; *p* = 0.342). The analysis of direct and indirect costs did not reveal statistically significant differences among the study groups (H = 3.34; *p* = 0.342 for both tests). Regarding the medians of direct costs, the results were as follows: the lowest costs were observed among patients diagnosed with FISH (€1186.03) and mPCR (€1393.62), followed by MALDI-TOF (€1660.94) and standard culture (€2015.84). The analysis of indirect costs demonstrated similar patterns: the lowest costs were recorded for patients diagnosed with FISH (€9912.97) and mPCR (€11,649.06), followed by MALDI-TOF (€13,880.28) and standard culture (€16,851.88) (Table 1).

### 2.4. Exploratory Adjusted Multivariable Analyses

To address potential residual confounding related to the retrospective observational design, additional exploratory multivariable analyses were conducted using parsimonious models. Covariates were selected a priori based on clinical relevance and included diagnostic method, age, sex, and severity category. These analyses were intended to complement, rather than replace, the primary nonparametric comparisons. Adjusted logistic regression demonstrated no independent association between diagnostic modality and mortality (all *p* > 0.05). Age remained the only significant predictor of mortality (OR = 1.03, 95% CI 1.01–1.06; *p* = 0.006), indicating a modest increase in risk with advancing age. Sex and severity category were not independently associated with mortality after adjustment. Negative binomial regression models evaluating total antibiotic-days similarly showed no independent effect of diagnostic method after adjustment (*p* = 0.206). The direction of effect remained consistent with the primary analyses, with rapid diagnostic methods demonstrating numerically higher antibiotic exposure, although this did not reach statistical significance. Neither age, sex, nor severity category were significantly associated with antibiotic utilization. Generalized linear models with gamma distribution and log link were applied to evaluate adjusted direct hospital costs. Diagnostic modality was not identified as an independent predictor of direct costs (*p* = 0.698), and no significant associations were observed for age, sex, or severity category. Overall, these adjusted analyses supported the findings of the univariable comparisons, suggesting that differences observed across diagnostic groups were not independently explained by basic demographic or clinical characteristics.

## 3. Discussion

This study compared three rapid microbiological diagnostic methods—multiplex PCR (mPCR), fluorescence in situ hybridization (FISH), and matrix-assisted laser desorption ionization–time-of-flight mass spectrometry (MALDI-TOF MS)—with the standard culture method for the diagnosis of bloodstream infections (BSIs). Although rapid methods are designed to accelerate identification and improve clinical outcomes, our findings indicate that their real-world clinical and economic impact is heterogeneous and largely dependent on integration with antimicrobial stewardship (AMS) practices.

During the study period, our institution used a consultative approach to real-time antimicrobial stewardship, coupled with rapid diagnostic tests. Rapid tests were selectively ordered by ICU physicians for patients with clinically suspected severe BSI requiring urgent clarification of the etiology. Upon request, testing was promptly performed by the microbiology laboratory, and results were proactively communicated by the clinical microbiologist to the ICU attending physician. Real-time discussion was provided regarding the identified microorganisms, their antimicrobial resistance, their therapeutic implications, and the microbiologist’s recommendations for escalation or de-escalation of therapy. It is important to note that this model was consultative and non-binding: prescribing authority remained with the ICU team, and automated protocol-driven antimicrobial modifications were not implemented. Furthermore, rapid diagnostic implementation was clinician-driven and not universal for all positive blood cultures. Therefore, the intensity of management depended on the urgency and availability of the case, and coverage was not structured as a fully standardized 24/7 prospective audit and feedback program. As new diagnostic platforms were successively introduced, workflows evolved and management integration varied across modalities. This nuanced implementation framework is critical for interpreting study results. The literature consistently shows that the clinical and economic benefits of rapid diagnostics are maximized when embedded in structured, protocolized AMS programs with standardized intervention pathways.

The higher antibiotic utilization observed in the mPCR group could theoretically reflect greater underlying patient complexity rather than a direct effect of the diagnostic modality. The diagnostic groups were not formed through random allocation but reflected real-world clinical practice during the sequential implementation of rapid diagnostic technologies. Standard culture was performed in all patients and served as the reference method throughout the study period. Rapid diagnostic tests (Multiplex PCR (FilmArray Blood Culture Identification Panel, BioFire Diagnostics/bioMérieux), Peptide Nucleic Acid Fluorescence In Situ Hybridization (PNA-FISH, QuickFISH BC, AdvanDx, Woburn, MA, USA), MALDI-TOF MS, VITEK MS, bioMérieux) were introduced progressively and were selectively applied following joint clinical–microbiological assessment. The decision to request a rapid test was initiated by the treating clinician based on clinical severity and perceived urgency, while the clinical microbiologist evaluated test feasibility, interpreted preliminary findings, and participated in therapeutic discussion once results became available. Thus, test utilization resulted from collaborative decision-making rather than protocolized assignment.

To determine whether increased antibiotic use in the mPCR group reflected baseline severity differences, we examined clinical severity indicators across diagnostic modalities. Rapid testing was more frequently considered in patients with suspected severe or very severe presentation; however, the overall distribution of severity categories did not differ significantly between rapid diagnostic and standard culture groups (very severe: 67.5% vs. 60.5%). Additionally, length of stay within severity strata showed no statistically significant differences, with overlapping interquartile ranges. These findings suggest that major structural differences in baseline clinical severity were unlikely to account for the increased antibiotic exposure observed in the mPCR cohort. Nevertheless, given the clinician-driven and consultative allocation process within a retrospective design, residual confounding by indication cannot be completely excluded.

In our cohort, the mPCR group had the highest empirical and total antibiotic-days and higher antibiotic-related costs versus FISH, MALDI-TOF, and standard culture (all significant). This diverges from several studies reporting reduced antibiotic exposure and earlier optimization with multiplex PCR when used alongside stewardship [14,15,16,17]. The discrepancy likely reflects implementation context (e.g., incomplete or non-embedded AMS workflows), which the literature repeatedly identifies as critical for realizing benefits from rapid tests [9,11]. Although several comparisons in our study did not reach statistical significance, clinically relevant numerical differences were observed across diagnostic groups. For instance, the median total antibiotic-days was highest in the mPCR group compared with FISH, MALDI-TOF, and standard culture, suggesting a potentially meaningful difference in antimicrobial exposure despite overlapping variability.

We observed no significant LOS difference across diagnostic strategies, with numerically lower medians for FISH and mPCR. This aligns with meta-analytic and trial data showing that while MALDI-TOF and PCR speed identification (by ~12–24 h for MAL-DI-TOF; 1–2 h for PCR), LOS reductions are modest and context-dependent [14,15,16,17,18,19,20,21]. FISH’s substantial time gains (>18 h for bacteria; >42 h for yeasts) also translate inconsistently to LOS without coordinated clinical response [22,23,24,25].

We found no association between diagnostic method and mortality (all ORs nonsignificant), which is consistent with large trials and reviews indicating limited or no mortality impact despite faster organism identification—particularly in the absence of robust stewardship and protocolized escalation/de-escalation pathways [11,17,18,19,20,21]. Given the sample size and observational design, mortality and length-of-stay analyses should be interpreted as exploratory, and the absence of statistical significance does not imply equivalence between diagnostic strategies.

Group comparisons for direct and indirect costs were not statistically different, though medians were lowest with FISH and mPCR. This partially echoes economic evidence: MALDI-TOF + AMS is most often cost-effective/dominant in models and trials [9,11,24], while PCR panels are frequently cost-effective with AMS but yield smaller absolute savings [9,25,26]. FISH is operationally simple and fast; its economic impact is less extensively modeled, yet our medians support its potentially favorable cost profile in re-al-world use [9,17,21]. In our study, direct and indirect cost medians were numerically lower in the FISH and mPCR groups compared with standard culture. These trends, while not statistically significant, may still reflect clinically relevant differences in resource utilization.

Finally, exploratory adjusted models confirmed the primary univariable findings, suggesting that diagnostic modality was not independently associated with mortality, antibiotic exposure, or direct hospital costs after accounting for basic clinical characteristics.

The test-specific economic positioning revealed the following:

1. MALDI-TOF MS: The literature supports strong cost-effectiveness with AMS (e.g., ≈$29,205/QALY; death averted per ~14 patients), but trial data (RAPIDO) show modest cost/survival differences when implementation varies [9,11,23]. Our neutral LOS/mortality results are concordant with this nuance.
2. Multiplex PCR: Generally cost-effective with stewardship (ICER ≈ $19.8k–$21k/QALY) and enables earlier optimization [9,12,17]. Our higher antibiotic-days and costs in the mPCR arm diverge from that pattern, again pointing to workflow/AMS integration effects rather than test performance per se.
3. FISH: Delivers rapid, reliable identification with large time gains [23,24]; modeling is less mature than for MALDI-TOF/PCR. Our lower cost medians with FISH are compatible with a cost-conscious role where infrastructure is limited or as an adjunct to other rapid methods.

Our results reinforce the literature’s central message: diagnostic speed ≠ clinical/economic benefit by default. The magnitude and direction of effects on antibiotics, LOS, and costs hinge on antimicrobial stewardship, local workflows, and case-mix—precisely the moderators highlighted across prior trials, reviews, and models [9,11,18,19,20,21,22,23,24,25,26].

The paradoxical increase in antibiotic exposure observed in the mPCR group represents a critical finding that diverges from the majority of the published literature and warrants careful interpretation. Most randomized controlled trials and prospective studies report that multiplex PCR, when coupled with antimicrobial stewardship, reduces broad-spectrum antibiotic use and accelerates de-escalation [17,27,28,29,30]. The discordance between our findings and these studies likely reflects implementation-dependent factors rather than inherent limitations of the technology itself. Several contextual explanations merit consideration. First, the consultative, non-binding nature of our stewardship model—wherein microbiologist recommendations were advisory rather than protocol-driven—may have resulted in inconsistent uptake of de-escalation opportunities, particularly during periods of high clinical acuity when prescribing inertia is greatest [31,32]. Second, the selective, clinician-initiated ordering of mPCR for perceived severe cases may have introduced behavioral confounding: physicians requesting rapid testing may have been predisposed to more aggressive antimicrobial management independent of test results, a phenomenon not captured by severity stratification alone [30,33]. Third, the absence of 24/7 availability and real-time audit and feedback mechanisms—features consistently associated with successful mPCR implementation in the literature—likely attenuated the technology’s potential to drive timely therapeutic optimization [17,31,34]. Banerjee et al. demonstrated that mPCR with templated comments alone reduced treatment of contaminants but required active stewardship involvement to achieve significant de-escalation (21 h vs. 38 h without stewardship, *p* < 0.001) [30]. Similarly, IDSA/SHEA guidelines emphasize that rapid diagnostics without concurrent ASP support often fail to improve antibiotic utilization or clinical outcomes [31]. Our findings thus underscore a critical principle: rapid diagnostic platforms are necessary but insufficient tools; their clinical value is contingent upon integration within structured, protocolized stewardship workflows with standardized intervention pathways and continuous availability [31,32,34,35].

### Strengths and Limitations

The main strength of this study is the combined clinical and economic evaluation of three rapid diagnostic modalities under routine conditions in a tertiary-care setting. The retrospective single-center design, partial lack of stewardship integration, and limited sample size represent constraints that may influence generalizability. The emphasis on effect direction and magnitude, rather than statistical significance alone, is particularly relevant in real-world observational cohorts where sample size may limit statistical power. Importantly, the distribution of 115 patients across four diagnostic groups may have reduced the ability to detect moderate differences in mortality, length of stay, and cost outcomes. Therefore, non-significant findings should be interpreted cautiously and should not be considered evidence of equivalence between diagnostic strategies. Furthermore, the economic component of the present study represents a cost comparison (cost consequence) analysis rather than a full cost-effectiveness evaluation, as no incremental modeling or utility-based outcomes were assessed. The wide confidence intervals observed for mortality further reflect potential imprecision related to clinical heterogeneity and sample size. In addition, the exploratory nature of subgroup analyses and variability in real-world antimicrobial stewardship practices may have influenced observed trends. Nevertheless, the study provides real-world evidence from a Central–Eastern European context, where cost data on rapid diagnostics remain scarce.

## 4. Materials and Methods

### 4.1. Study Design and Setting

A retrospective observational study was conducted over 5 years and 8 months (January 2015–August 2020) at the Department of Medical Microbiology and Immunology “Prof. Dr. Elissay Yanev” and the Innovative Diagnostic Methods Unit of the Research Institute, Medical University–Plovdiv, in collaboration with the Microbiology Laboratory of University Hospital “St. George,” Plovdiv.

### 4.2. Study Population

The analysis included 115 patients hospitalized at the Clinic of Anesthesiology and Intensive Care of University Hospital “St. George.” Eligible patients had suspected or confirmed bloodstream infections (bacteremia or fungemia) and met predefined inclusion and exclusion criteria. Inclusion criteria: (1) Patients of any age or sex with ≥2 clinical indicators of systemic infection (e.g., fever >38 °C or <36 °C, leukocytosis >12 × 10^3^/µL or leukopenia <4 × 10^3^/µL, tachycardia >90 bpm, tachypnea >20 breaths/min, or PaCO_2_ < 32 mm Hg). (2) Blood cultures obtained before or ≥24 h after antibiotic administration. Exclusion criteria: (1) Sampling without aseptic conditions. (2) Single-bottle blood culture in adults. (3) Only one or no clinical indicator of infection.

Rapid diagnostic modalities (Multiplex PCR (FilmArray Blood Culture Identification Panel, BioFire Diagnostics/bioMérieux), Peptide Nucleic Acid Fluorescence In Situ Hybridization (PNA-FISH, QuickFISH BC, AdvanDx, Woburn, MA, USA), MALDI-TOF MS, VITEK MS, bioMérieux) were introduced sequentially as they became available and validated for clinical implementation. Patients were not randomly assigned to diagnostic methods. The decision to perform rapid testing was initiated by the treating clinician based on clinical presentation, perceived severity, urgency, and anticipated impact on management. Platform availability at the time of testing also influenced allocation.

Thus, diagnostic modality assignment reflected real-world clinical workflow and temporal implementation rather than predefined protocol criteria. This pragmatic allocation approach introduces the potential for selection and temporal bias, which is acknowledged in the interpretation of the results.

### 4.3. Cost Components

Hospital Bed-Day Costs: The average cost per hospital bed-day was calculated using data from the accounting system of University Hospital “St. George.” The system allocates direct and indirect costs according to Bulgarian accounting standards. Direct costs include food for inpatients, medical supplies, blood and blood products, bioproducts, implants, and laboratory services. Indirect costs cover utilities, maintenance, external services, depreciation, staff salaries, social security contributions, taxes, and other administrative expenses. All monthly expenses were summed to determine the average cost per bed-day for each patient, expressed separately as direct and indirect components.

Antibiotic Therapy Costs: Drug-related costs were extracted from direct hospital expenditures. For each patient, antibiotic costs were calculated based on the administered dose and unit price per day (“antibiotic-day”), using data from the hospital information system.

Diagnostic Test Costs: Laboratory costs were included in direct expenses, while micro-biological test costs (rapid or culture-based) were also reported separately for clarity.

Clinical Pathways and Procedures: Costs for clinical pathways (CPs) and clinical procedures (CPrs) were calculated using official tariffs from the National Framework Agreements between the National Health Insurance Fund and the Bulgarian Medical Association (2017–2019).

For procedures (CPr 3 and CPr 4), costs were estimated as a proportion of the total bed-days during which they were applied. Since these procedures are billed per 24 h and cannot overlap, their combined share (89.51%) was applied to individual patient data.

The economic evaluation was conducted as a cost comparison (cost consequence) analysis describing differences in resource utilization and costs across diagnostic strategies; no formal cost-effectiveness modeling or incremental cost-effectiveness ratios were performed. All cost data were initially collected and calculated in Bulgarian lev (BGN) based on hospital accounting records and national reimbursement tariffs. For improved international comparability and readability, cost values were converted to euros (EUR) using the fixed exchange rate established under Bulgaria’s currency board arrangement (1 EUR = 1.95583 BGN). This exchange rate has remained stable for many years under the international monetary framework governing the Bulgarian monetary system; therefore, no additional currency adjustment or temporal correction was applied during the study period. The conversion was performed uniformly across all direct and indirect cost categories.

### 4.4. Statistical Methods

Quantitative variables with normal distribution were expressed as mean ± standard deviation (SD), while non-normally distributed variables were presented as median and interquartile range (25th–75th percentiles). Qualitative variables were summarized as frequencies and percentages (n, %). Primary analyses were conducted using nonparametric statistical approaches due to the skewed distribution of clinical and economic outcomes. The Chi-square (χ^2^) test was used to assess associations between categorical variables. Differences between independent groups were evaluated using the Kruskal–Wallis H test, and ordered alternatives were explored using the Jonckheere–Terpstra test where appropriate. Pairwise subgroup comparisons were interpreted based on the corresponding adjusted test statistics provided by the applied nonparametric procedures, as implemented in SPSS (version 26.0), and no additional external multiplicity correction was performed. A *p*-value < 0.05 was considered statistically significant. To further explore potential residual confounding inherent to the retrospective observational design, additional exploratory multivariable analyses were performed using parsimonious models. Logistic regression was applied to evaluate associations between diagnostic modality and mortality. Negative binomial regression models were used to analyze antibiotic-days due to the overdispersed count distribution of these outcomes. Generalized linear models (GLMs) with gamma distribution and log link were applied for cost outcomes to account for right-skewed economic data. All multivariable models included diagnostic method, age, sex, and severity category as covariates, with model complexity intentionally limited to minimize overfitting given the sample size. All analyses were conducted using IBM SPSS Statistics for Windows, version 26.0 (Armonk, NY, USA: IBM Corp.).

## 5. Conclusions

Rapid diagnostic technologies—MALDI-TOF MS, multiplex PCR, and FISH—demonstrably shorten time to pathogen identification compared to conventional culture. However, their downstream impact on antibiotic use, hospital costs, and outcomes is variable. Our findings, in line with international evidence, suggest that the full clinical and economic value of rapid diagnostics is realized only when integrated within robust antimicrobial stewardship programs. Future multicenter studies should further quantify these effects and define context-specific implementation models to optimize both resource allocation and patient benefit.

## Figures and Tables

**Table 1:** Comparison of patient characteristics across study groups—subgroups of rapid diagnostic tests versus standard culture (*n* = 115).

Variables	Rapid Diagnostic Tests (*n* = 77)			Standard Culture-Based Method (*n* = 38)	*p-Value*
	mPCR (*n* = 27)	FISH (*n* = 25)	MALDI-TOF (*n* = 25)		
*Demographic characteristics*					
Males, *n (%)*	15 (55.56)	17 (68.00)	19 (76.00)	29 (76.32)	0.279 ^1^
Age (years), *mean ± SD*	56.59 (48, 69)	52 (43, 62)	45 (22, 60)	51 (30, 60)	0.071 ^2^
*Clinical Data*					
Length of hospital stay (days), median [25th–75th percentile]	31; 17, 43	15; 8, 27	20; 12, 31	16; 10, 31	0.071 ^2^
Antibiotic-days—empirical therapy (days), median [25th–75th percentile]	22; 13, 40	10; 7, 15	10; 6, 20	14; 8, 20	0.004 ^2^
Antibiotic-days—targeted therapy (days), median [25th–75th percentile]	12; 5, 33	8; 5, 20	16; 3, 28	6; 1, 18	0.263 ^2^
Antibiotic-days—total (days), median [25th–75th percentile]	47; 21, 59	19; 13, 38	28; 11, 45	23; 12, 32	0.012 ^2^
Change in therapy after diagnostic test result—yes, *n* (%)	19 (70.37)	14 (56.00)	17 (68.00)	22 (57.89)	0.608 ^1^
Changes in therapy following diagnostic test result, *n* (%):					
Died before result	4 (14.81)	4 (16.00)	6 (24.00)	9 (23.68)	0.477 ^3^
Continued the same antibiotic therapy	4 (14.81)	7 (28.00)	3 (12.00)	7 (18.42)	
Discontinued antibiotic therapy	1 (3.70)	0 (0.00)	0 (0.00)	0 (0.00)	
Changed antibiotic therapy	18 (66.67)	14 (56.00)	16 (64.00)	22 (57.89)	
Lethality, *n* (%)	14 (51.85)	16 (64.00)	12 (48.00)	21 (55.26)	0.701 ^1^
*Costs*					
Direct costs (€), median [25th–75th percentile]	1838.14; 1008.01, 2549.70	889.44; 474.39, 1630.62	1186.03; 711.54, 1838.14	948.73; 592.97, 1838.14	0.071 ^2^
Indirect costs (€), median [25th–75th percentile]	15,365.17; 8426.45, 21,314.90	7434.10; 3965.31, 13,630.58	9912.97; 5948.86, 15,365.17	7930.81; 4956.67, 15,365.17	0.071 ^2^
Costs of clinical pathway (€), median [25th–75th percentile]	1586.04; 613.55, 2193.42	1662.75; 587.99, 2103.44	1670.39; 884.02, 2935.17	1248.58; 429.01, 1670.39	0.112 ^2^
Costs of clinical procedure (€), median [25th–75th percentile]	8277.07; 4538.09, 11,481.89	4005.00; 2136.05, 2229.69	5339.19; 3204.05, 8277.07	4271.93; 2669.01, 8277.07	0.071 ^2^
Costs of diagnostic tests (€), median [25th–75th percentile]	204.56; 204.56, 204.56	28.13; 28.13, 28.13	1.02; 1.02, 1.02	19.43; 19.43, 19.43	0.000 ^2^
Antibiotic therapy costs—total (€), median [25th–75th percentile]	637.67; 379.83, 1077.52	256.39; 67.32, 510.74	398.69; 127.51, 758.46	301.66; 104.80, 716.61	0.047 ^2^
Antibiotic therapy costs—empirical (€), median [25th–75th percentile]	323.26; 139.91, 669.69	117.22; 37.53, 252.79	142.66; 48.84, 257.87	120.72; 50.36, 271.91	0.016 ^2^
Antibiotic therapy costs—targeted (€), median [25th–75th percentile]	173.28; 31.35, 564.81	88.78; 13.89, 369.34	271.40; 3.21, 615.40	131.11; 11.31, 382.85	0.657 ^2^

^1^ Chi-square (χ^2^) test; ^2^ Kruskal–Wallis H test; ^3^ Jonckheere–Terpstra, JT.

## Data Availability

Research data can be obtained upon request.

## Abbreviations

The following abbreviations are used in this manuscript:

ASPs	Antimicrobial stewardship programs
BSIs	Bloodstream infections
CPs	Clinical pathways
CPrs	Clinical procedures
ICU	Intensive care unit
mRDTs	Molecular rapid diagnostic tests
RDTs	Rapid diagnostic tests
SD	Standard deviation
